# All-in-One Nanohybrids Combining Sonodynamic Photodynamic and Photothermal Therapies

**DOI:** 10.1021/acsami.4c09715

**Published:** 2024-08-13

**Authors:** Dilsad Taydas, Muhammed Emre Özler, Mustafa Ergül, Zeynep Deniz Şahin İnan, Fazlı Sözmen

**Affiliations:** † Nanotechnology Engineering Department, Faculty of Engineering, Sivas Cumhuriyet University, Sivas 58140, Türkiye; ‡ Biochemistry Department, Faculty of Pharmacy, Sivas Cumhuriyet University, Sivas 58140, Türkiye; § Histology and Embryology Department, Faculty of Medicine, Sivas Cumhuriyet University, Sivas 58140, Türkiye

**Keywords:** hollow nanohybrids, photodynamic therapy, sonodynamic therapy, photothermal therapy, thermosensitive polymer

## Abstract

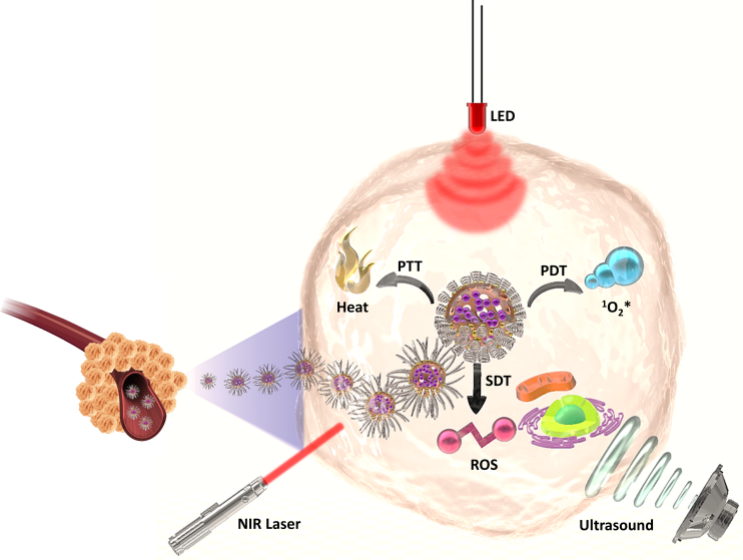

A wide variety of methods are being developed to ultimately defeat cancer; while some of these strategies have shown highly positive results, there are serious obstacles to overcome to completely eradicate this disease. So, it is crucial to construct multifunctional nanostructures possessing intelligent capabilities that can be utilized to treat cancer. A possible strategy for producing these multifunctional nanostructures is to combine various cancer treatment techniques. Based on this point of view, we successfully synthesized multifunctional HCuS@Cu_2_S@Au–P(NIPAM-*co*-AAm)-PpIX nanohybrids. The peculiarities of these thermosensitive polymer-modified and protoporphyrin IX (PpIX)-loaded hollow nanohybrids are that they combine photodynamic therapy (PDT), sonodynamic therapy (SDT), and photothermal therapy (PTT) with an intelligent design. As an all-in-one nanohybrids, HCuS@Cu_2_S@Au–P(NIPAM-*co*-AAm)-PpIX nanohybrids were employed in the SDT–PDT–PTT combination therapy, which proved to have a synergistic therapeutic effect for in vitro tumor treatments against breast tumors.

## Introduction

Each cancer therapy has its restrictions, and overcoming these issues is crucial to improving the effectiveness of the therapy. One or more therapy methods may be employed concurrently for treating cancer.^1^ Numerous variables, including the type of cancer, pathology, tumor size, and degree of dissemination, influence the sort of treatment that should be given. Various treatment modalities such as chemotherapy, radiotherapy, photodynamic therapy (PDT), photothermal therapy (PTT), and sonodynamic therapy (SDT) have been applied to induce apoptosis or immunogenic cell death.^2−5^ There is no definitive treatment for many types of cancer, even though some treatments have had very positive results. This result forced us, like many researchers, to develop novel and distinctive designs.

Light, a photosensitizer, and molecular oxygen are used specially in PDT. While PDT has been used successfully in the treatment of acne, skin lesions, and some eye diseases, it has been seen as a promising treatment for cancer.^6−9^ However, PDT could not show the expected effect due to its shortcomings, such as light penetration and a supporting hypoxic medium. To overcome the shortcomings of PDT related to the hypoxic medium, molecular oxygen-delivering systems or compounds that produce singlet oxygen independently of the molecular oxygen of the medium, such as endoperoxides, have been developed.^10,11^ Nevertheless, PDT has not yet reached the expected level of success in cancer treatment.

PTT is another NIR light-based therapeutic approach. PTT operates by generating localized heat with a specific wavelength of light and a photothermal agent to ablate tumor tissues selectively.^12^ Low toxicity, high biocompatibility, long circulation, improved tumor accumulation, efficient absorption of NIR light, and high efficiency heat conversion are all the desired features for a successful photothermal therapeutic outcome.^13^ Although various studies have been conducted to meet these features, the desired success in cancer treatment has not been achieved with PTT alone.

SDT has evolved as an alternative therapeutic strategy using low-intensity ultrasound (US) and a sonosensitizer to generate reactive oxygen species (ROS).^14^ One of the significant drawbacks of noninvasive phototherapies is the low penetration of the light utilized to activate the photosensitizer into the skin. However, sonodynamic therapy uses a high-frequency mechanical sound wave (1–3 MHz) to reach deeper tumors. Because of the deep penetration of US, it is widely employed in hospitals for diagnostic imaging, and has the potential to be highly beneficial in the activation of sonosensitizers. Recently, it has become quite common to use sonosensitizers as encapsulated nanostructures since they are less affected by physiological conditions and increase SDT efficiency.^15^ SDT, however, is also insufficient on its own to address the issues associated with cancer therapy.

Due to their NIR localized surface plasmon resonance (LSPR) absorption and high photothermal conversion efficiencies, copper sulfide (CuS) nanoparticles and cuprous sulfide nanostructures with copper-deficient stoichiometries (Cu_2-*x*_S) have been the focus of several studies.^16^ However, the LSPR coupling effect between copper sulfide derivatives and noble metals can cause an increase in the photothermal effect when copper sulfide derivatives are combined with a noble metal, such as gold, as in gold–copper sulfide nanocomposites, gold–copper sulfide core–shell nanoparticles, and gold–copper sulfide hybrid nanosystems.^2,17^

When designing combined therapeutic nanostructures, it is crucial to protect the nanostructures from physiological conditions and make them functional at the desired place and moment. In this regard, biocompatible smart polymers are highly preferred as an important option.^18,19^ In particular, designing heat- or light-sensitive smart polymers and incorporating them into such stimuli-activated nanostructures enable them to function as intended. The solubility of thermosensitive polymers may suddenly change in response to a slight temperature change. The temperature at which a sudden change in this solubility of a polymer and total volume occurs is generally known as the cloud point.^20,21^ Aqueous thermosensitive polymer solutions exhibit various cloud points depending on the polymer chain’s ratio of hydrophilic and lipophilic constituents. These polymers can be evaluated in two main groups that exhibit two distinct dissolving temperature parameters. Of these polymers with a lower critical solution temperature (LCST), the polymer system is soluble below the critical temperature. However, above the critical temperature, the polymer system becomes more hydrophobic and less soluble, and phase separation is observed with clouding. A polymer having an upper critical solution temperature (UCST), on the other hand, operates in the opposite way.^22,23^ Poly-*N*-isopropylacrylamide (PNIPAM) is one of the most used LCST thermosensitive polymers, and the phase diagram of PNIPAM indicates that the LCST value is around 32 °C.^24^ By copolymerizing with a hydrophilic monomer, the LCST temperature of PNIPAM can be raised to a mild hyperthermia temperature (43 °C).^19^ Each approach or material we have discussed so far has both pros and cons in cancer treatment. Hence, we chose the all-in-one concept and intended to combine all of these methods and materials. Thus, we synthesized HCuS@Cu_2_S@Au–P(NIPAM-*co*-AAm)-PpIX nanohybrids, which were enhanced with P(NIPAM-*co*-AAm) (poly(*N*-isopropylacrylamide-*co*-acrylamide)), a thermosensitive copolymer, and loaded with PpIX (protoporphyrin IX), an agent for SDT and PDT. This innovative approach was designed for multifaceted cancer therapy. Consequently, these hybrid nanostructures were shown to have a greater anticancer effect when simultaneously treated with laser (808 nm, 1.5 W/cm^2^), US (3 MHz), and LEDs (630 nm) than either individually or in dual combinations in in vitro cell investigations.

## Results and Discussion

**Scheme 1:**
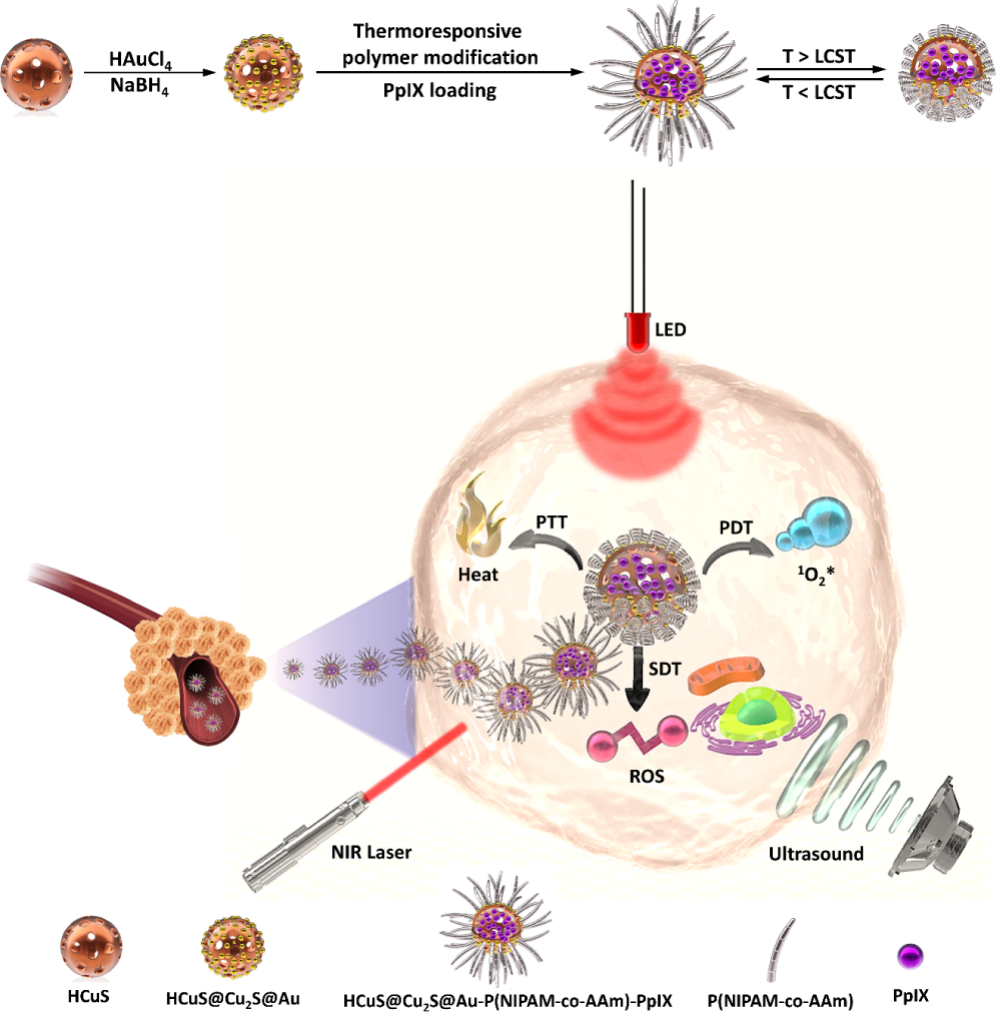
Schematic Illustration of the Synthesis Procedures and SDT–PDT–PTT Triple Therapy Effect

This work started with the synthesis of cleverly designed HCuS@Cu_2_S@Au–P(NIPAM-*co*-AAm)-PpIX hollow nanohybrid structures to realize three therapeutic modalities on a single platform. The triple therapy effect with the synthesis procedure is schematically shown in Scheme 1. At first, hollow-structured CuS (HCuS) nanoparticles were synthesized by a one-pot sacrificial template method according to the literature.^17^ The size distribution and zeta potential of HCuS nanoparticles were measured by dynamic light scattering (DLS) at 343 nm and −12 mV, respectively (Figures S1and 4^4^F). In addition, SEM–EDS analysis of HCuS nanoparticles also supports the size and hollow structure. While the formation of HCuS nanoparticles was also confirmed by X-ray diffraction (XRD) analysis, the XRD patterns are highly consistent with the literature for CuS (Figure 2F).^17,25^

**Figure 1:**
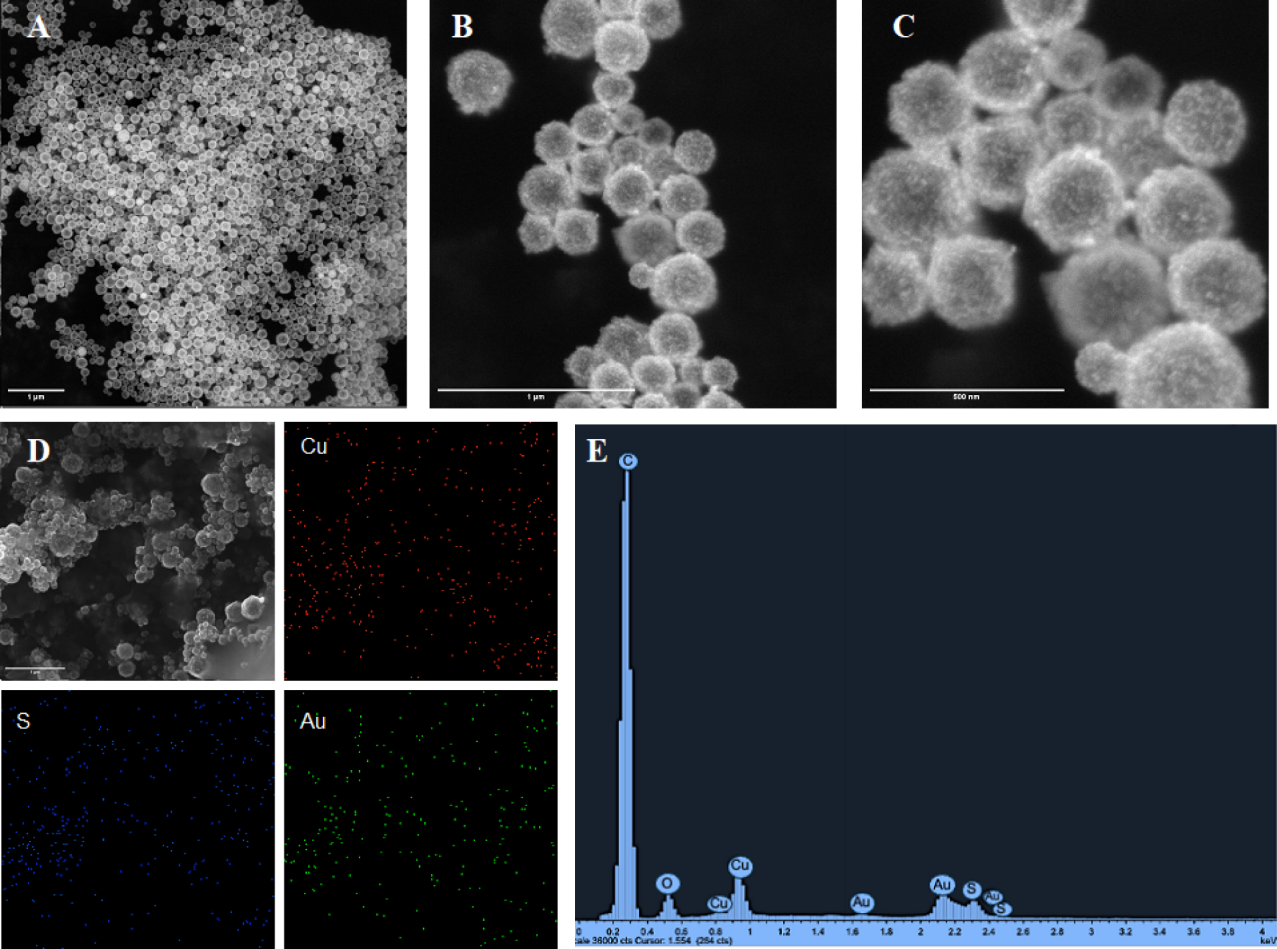
SEM images of HCuS (A), HCuS@Cu_2_S@Au (B,C), EDS elemental mapping (D), and EDS characterization of HCuS@Cu_2_S@Au (E).

**Figure 2:**
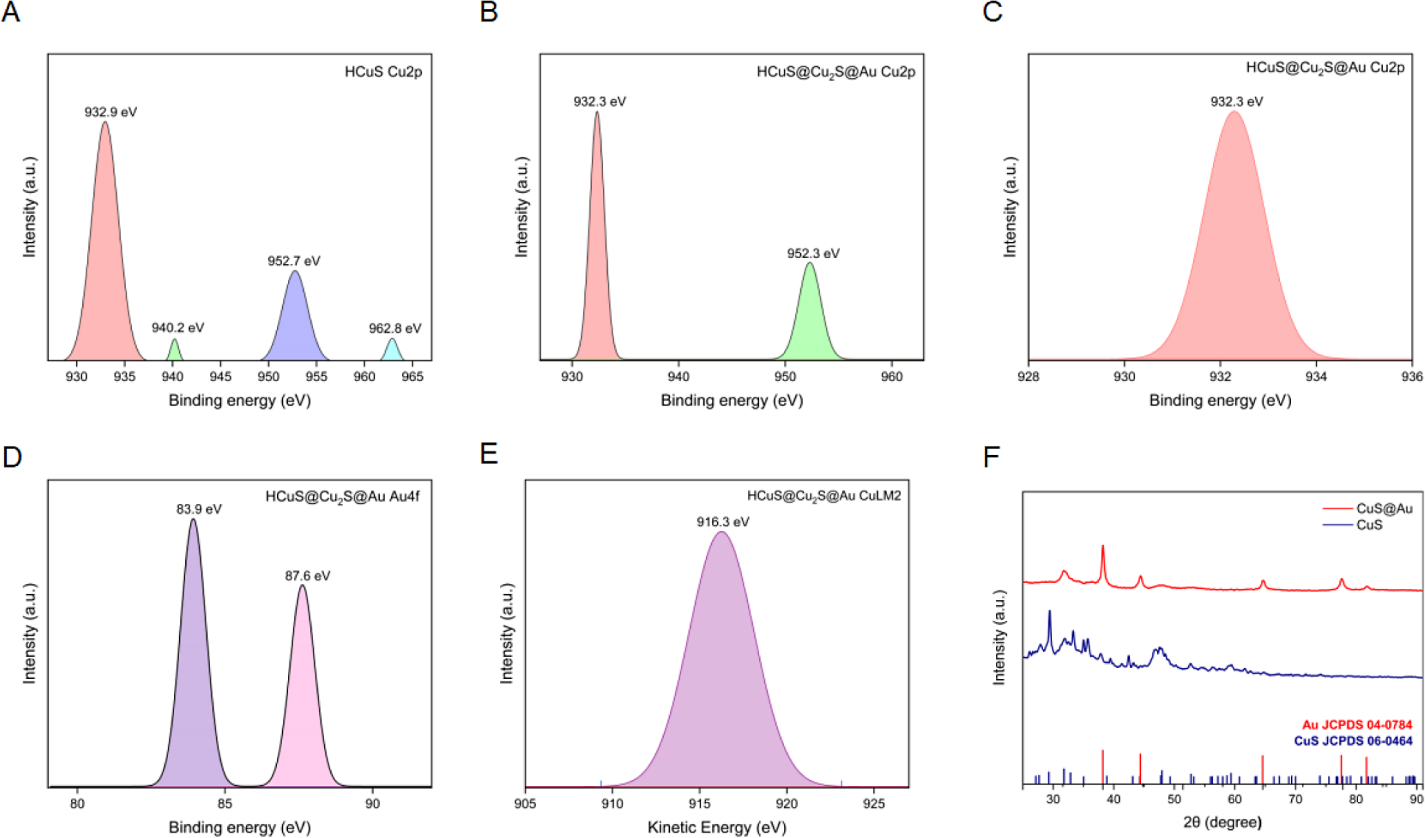
XPS high-resolution scans of Cu 2p peaks in HCuS (A) and HCuS@Cu_2_S@Au (B). Detailed spectra of Cu 2p_3/2_ in HCuS@Cu_2_S@Au (C). XPS high-resolution scans of Au 4f in HCuS@Cu_2_S@Au (D) and Auger electron spectrum of HCuS@Cu_2_S@Au CuLM2 (E). XRD patterns of CuS and CuS@Au (F).

In 2017, Deng et al. synthesized hollow structured HCuS@Cu_2_S@Au nanohybrids and published an article on photoswitchable targeting effects for cancer theranostics.^17^ We also synthesized and characterized these hollow structured HCuS@Cu_2_S@Au nanohybrids, which have an enhanced PTT effect, from HCuS nanoparticles. The formation of nanoshell/satellite-structured HCuS@Cu_2_S@Au nanohybrids obtained by uniform deposition of spherical gold nanoparticles on the surface of HCuS nanoparticles was also observed by us with a scanning electron microscope (SEM) (Figure 1). Moreover, the agreement of the XRD analysis results of HCuS nanoparticles and HCuS@Cu_2_S@Au nanohybrids with the hexagonal phase CuS (JCPDS No. 06–0464) and cubic phase Au (JCPDS No. 04–0784) standard data supports the formation of CuS and CuS@Au (Figure 2).^17^ In addition, in agreement with the literature,^17,25^ while four Cu 2p peaks (2p_3/2_, 2p_1/2_ peaks, and their satellite peaks) are seen in the XPS (X-ray photoelectron spectroscopy) spectra of Cu(II)S hollow nanoparticles, two peaks of Cu (2p_3/2_ and 2p_1/2_ peaks) are observed in the XPS spectrum of CuS@Au hollow nanohybrids (Figure 2). Moreover, when the modified Auger parameter for Cu, indicating the summation of photoelectron binding energy and Auger kinetic energy, was calculated to be 916.3 eV, the existence of Cu(I) on CuS@Au was confirmed. Additionally, the presence of Cu, S, and Au was verified by energy dispersive X-ray (EDS) analysis for CuS@Cu_2_S@Au nanohybrids (Figure 1). Furthermore, using inductively coupled plasma mass spectrometry (ICP-MS) analysis, the Cu and Au contents in CuS@Cu_2_S@Au nanohybrids were found to be 27.871% and 72.129%, respectively. The N_2_ adsorption and desorption isotherms of HCuS@Cu_2_S@Au are also presented in Figure S2. The nanohybrids’ isotherms, which are compatible with type IV isotherms with H1 hysteresis loops, point to a hollow, mesoporous structure.^26^ The total pore volume and BET (Brunauer, Emmett, and Teller) surface area of the CuS@Cu_2_S@Au nanohybrids were determined to be 0.267 cm^3^ g^–1^ and 37.2 m^2^ g^–1^, respectively.

**Figure 3:**
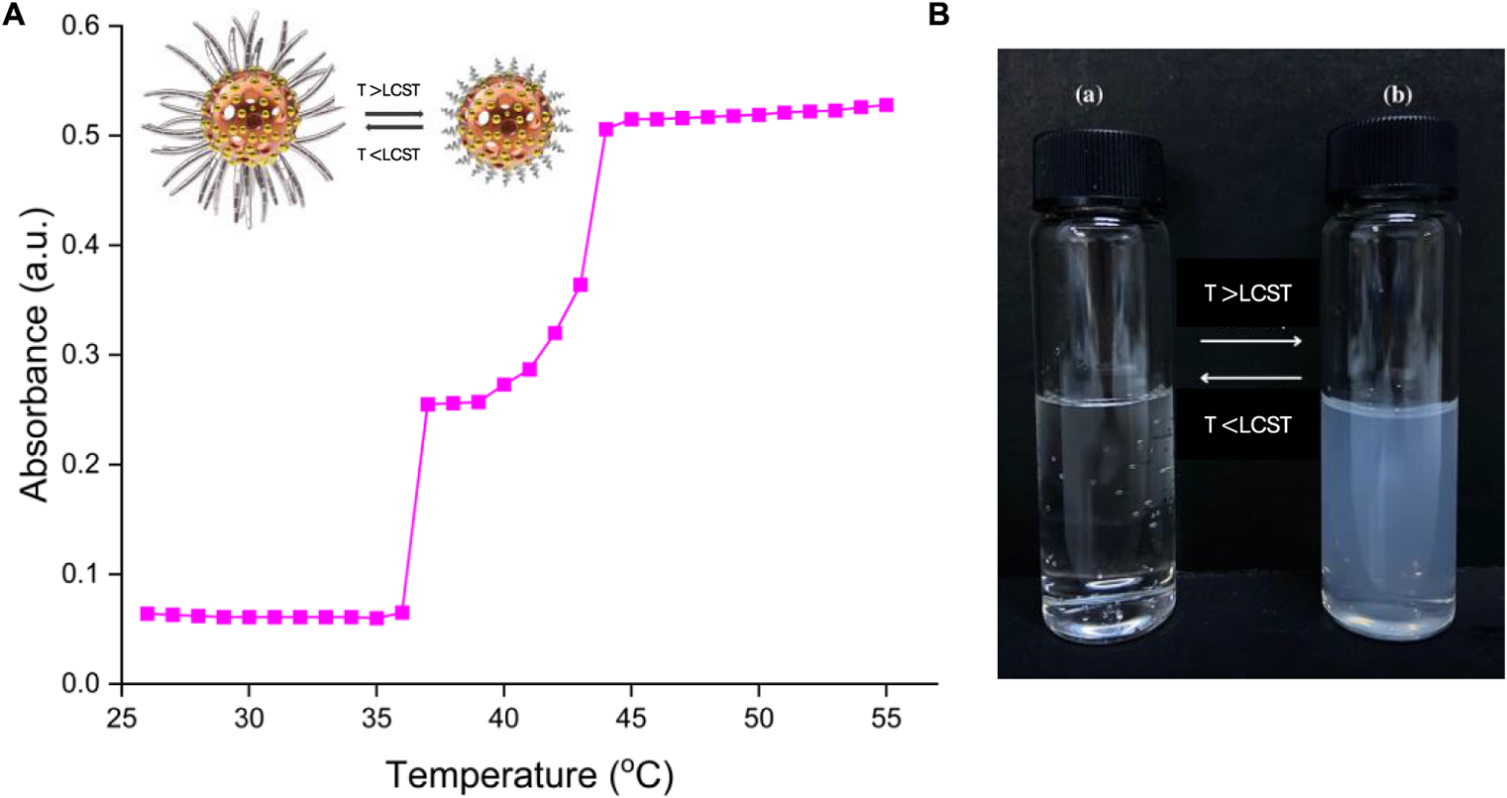
LCST measurement of P(NIPAM-*co*-AAm) copolymer’s aqueous solution (A) and LCST behavior of P(NIPAM-*co*-AAm) in aqueous solution (B). (a) below the cloud point and (b) above the cloud point, the solution became turbid.

For thermosensitive polymer modification of HCuS@Cu_2_S@Au nanohybrids, we needed a thermoresponsive polymer with a suitable LCST value, and PNIPAAm comes first among the polymers that come to mind when a thermosensitive polymer is mentioned. However, the LCST value of this thermosensitive polymer is about ∼30–32 °C.^20^ The LCST value can be tuned by obtaining PNIPAAm as a copolymer with a hydrophobic or hydrophilic group. Generally, PNIPAAm is copolymerized with acrylamide and the LCST value is increased, thus working at temperatures compatible with the physiological temperature of 37 °C.^19,20,24^ As a result, P(NIPAM-*co*-AAm) was synthesized by RAFT polymerization according to the literature and characterized by ^1^H NMR and FTIR spectroscopy (Figures S3and 4^4^E). The obtained results are highly compatible with the literature.^23,27^ For an LCST polymer, when the temperature of the solution surpasses the transition temperature, the copolymer aggregates and exhibits a cloudy appearance or phase separation of the solution. Conversely, when the temperature of the solution is reduced below the LCST, the interaction between water and the polymer intensifies due to hydrogen bonding, leading to a transparent solution. However, when the temperature exceeds the LCST, the hydrophobic interactions between the polymers increase and the hydrogen bonds break. This results in the formation of polymer globules, making the solution cloudy (Figure 3B). Determining the lower critical solution temperature (LCST) of a specific polymer solution through the cloud point method needs to be standardized. The cloud point, which some consider a 90% transmittance, while others at 50% or even 10%, can vary. In our research, we defined the cloud point at 50% transmittance.^20^ The LCST value of the P(NIPAM-*co*-AAm) copolymer synthesized in this study by RAFT polymerization was determined by the turbidimetric analytical method. The turbidity of the copolymer solution was measured at 500 nm with a microplate reader (Thermo). The polymer solution (0.1 wt %) was prepared in distilled water and the UV absorbances were measured from 26 to 55 °C, and the readings were taken after 5 min equilibration at each temperature. The polymer solution exhibited a rapid change in absorption in the 38–45 °C temperature range. The LCST of P(NIPAM-*co*-AAm) was measured to be 44 °C (Figure 3A).

**Figure 4:**
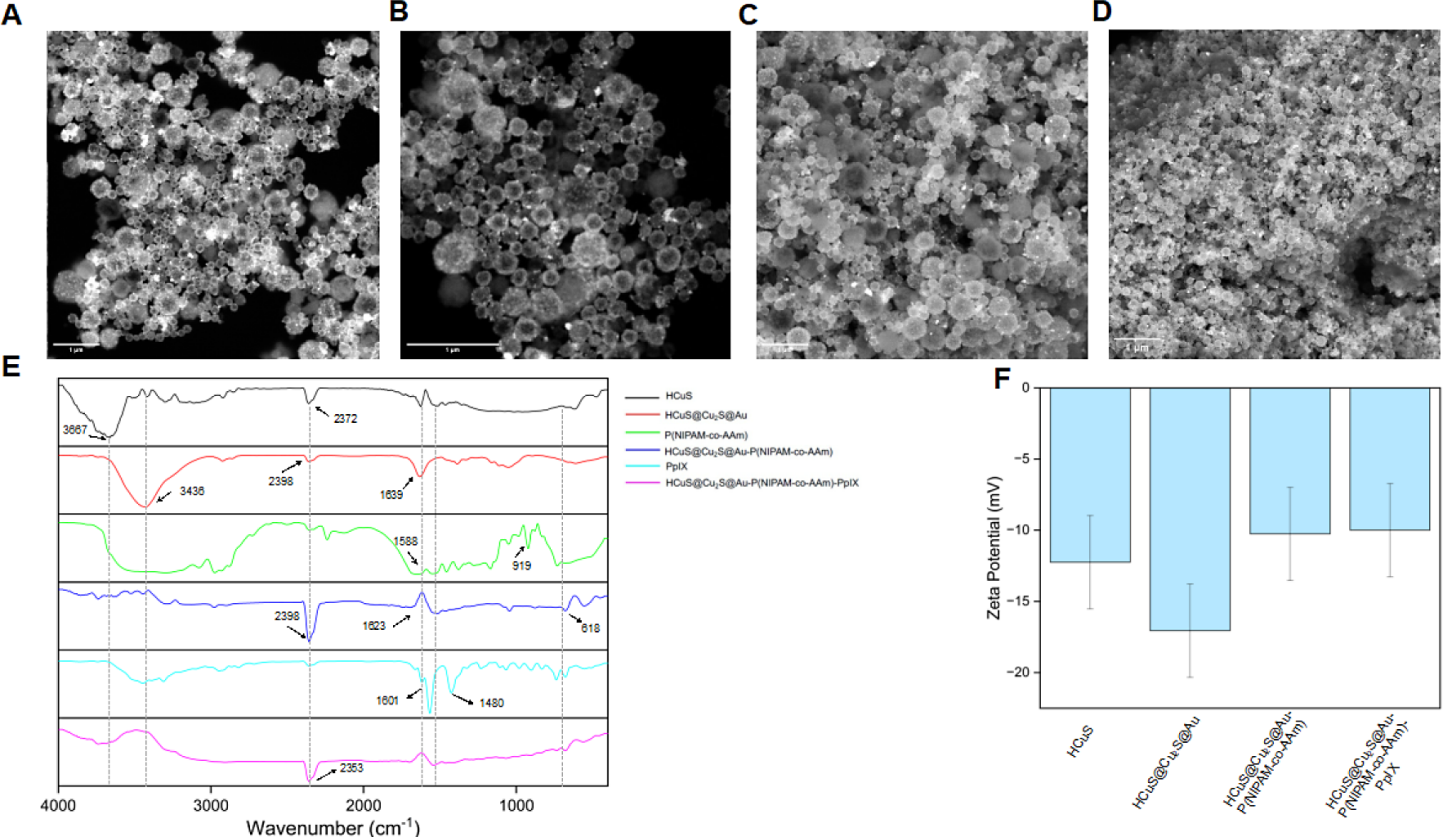
SEM images of HCuS@Cu_2_S@Au–P(NIPAM-*co*-AAm) (A,B) and HCuS@Cu_2_S@Au–P(NIPAM-*co*-AAm)-PpIX (C,D). All scale bars are 1 μm. FTIR spectra of HCuS, HCuS@Cu_2_S@Au, P(NIPAM-*co*-AAm), and HCuS@Cu_2_S@Au–P(NIPAM-*co*-AAm) (E) and zeta potentials of HCuS, HCuS@Cu_2_S@Au, HCuS@Cu_2_S@Au–P(NIPAM-*co*-AAm), and HCuS@Cu_2_S@Au–P(NIPAM-*co*-AAm)-PpIX (F).

Then, the hollow HCuS@Cu_2_S@Au nanohybrids were modified according to the literature with the P(NIPAM-*co*-AAm) copolymer with a more suitable LCST value for physiological conditions.^17^ The SEM images of these polymer-modified HCuS@Cu_2_S@Au–P(NIPAM-*co*-AAm) nanohybrids are given in Figure 4 and the average particle sizes were determined as 354 nm in the measurements performed with DLS (Table S1). SEM images and FTIR analysis results also support the idea that HCuS@Cu_2_S@Au nanohybrids are modified with thermosensitive polymer P(NIPAM-*co*-AAm) (Figure 4). In the final stage of the design, polymer-modified HCuS@Cu_2_S@Au–P(NIPAM-*co*-AAm) nanohybrids were loaded with PpIX, which has both sonosensitizing and photosensitizing effects.^18^ The SEM images and FTIR spectra of HCuS@Cu_2_S@Au–P(NIPAM-*co*-AAm)-PpIX nanohybrids indicate that polymer-modified and PpIX-loaded hollow HCuS nanohybrids were successfully synthesized (Figure 4). When the FTIR spectra in Figure 4E are examined in more detail, even though some peaks cover other peaks, the spectrum of HCuS@Cu_2_S@Au–P(NIPAM-*co*-AAm)-PpIX final nanohybrids contains nearly all of the peaks corresponding to all species present in the nanohybrids’ structure. Furthermore, as shown below, PDT and SDT measurements also validated the loading of PpIX into copolymer-modified HCuS@Cu_2_S@Au–P(NIPAM-*co*-AAm) nanohybrids. It appears that the sizes and zeta potentials of all nanostructures, including the final HCuS@Cu_2_S@Au–P(NIPAM-*co*-AAm)-PpIX nanohybrids, are between 343 and 427 nm, and −17.066 and −9.996 mV, respectively (Table S5). This means that an average size range and a zeta potential range consistent with the literature are preserved in all syntheses,^2,17,18^ Thus, the synthesized HCuS@Cu_2_S@Au–P(NIPAM-*co*-AAm)-PpIX nanohybrids emerged as all-in-one nanohybrid structures with the triple synergistic effects of SDT, PDT, and PTT.

**Figure 5:**
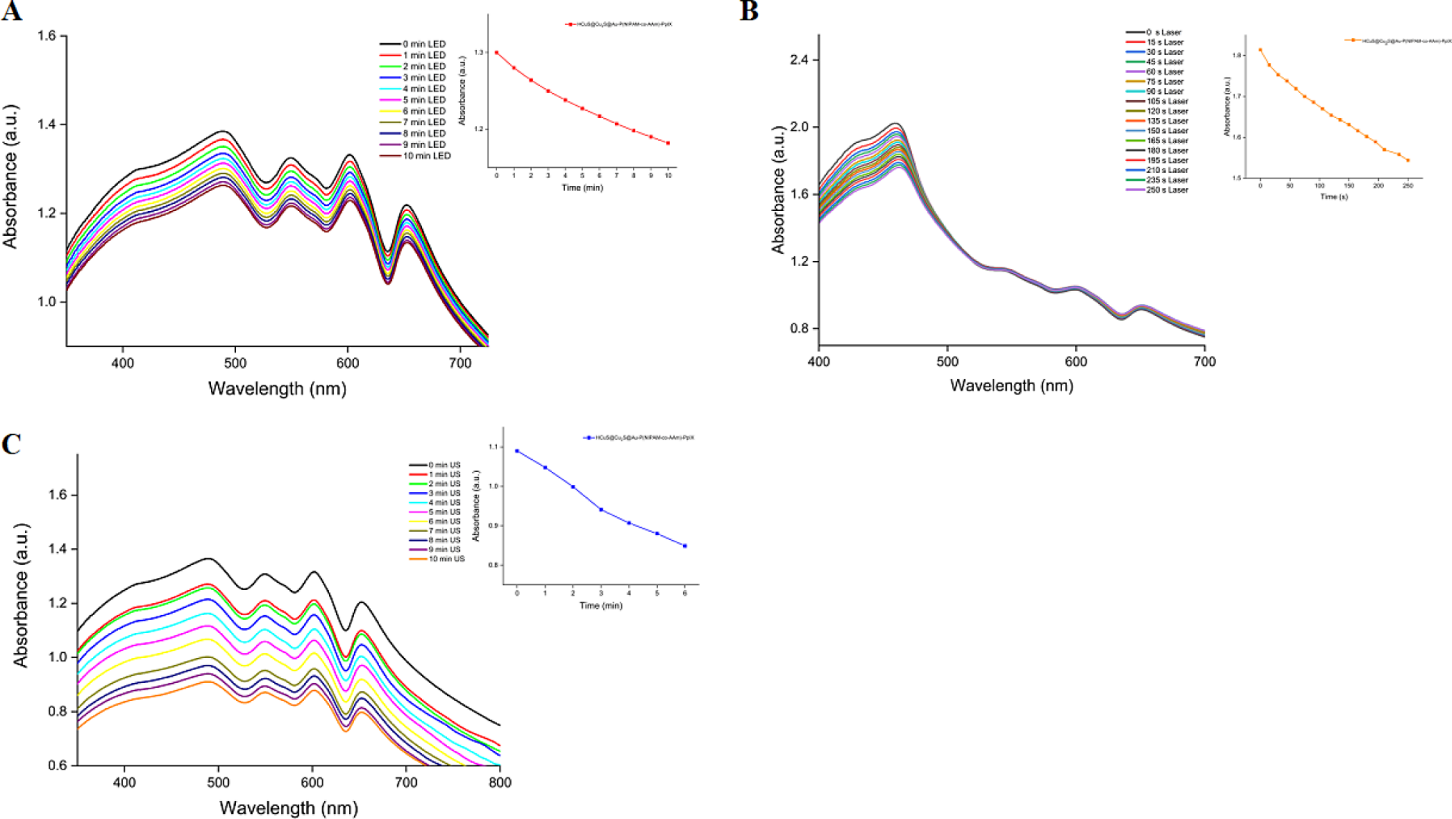
Decrease in the absorbance of DPBF in the presence of 0.8 mg/mL of hybrid nanoparticles at 416 nm in PBS/DMSO after irradiation with a 630 nm LED (A). Decrease in the absorbance of DPBF in the presence of 0.8 mg/mL of hybrid nanoparticles at 416 nm in PBS/DMSO after irradiation with 808 nm (1.5 W/cm^2^) laser (B). Decrease in the absorbance of DPBF in the presence of 0.8 mg/mL of hybrid nanoparticles at 416 nm in PBS/DMSO after irradiation with 3 MHz US (C). Insets: graphs showing the decrease in the maximum absorbance of DPBF at 416 nm over time in the presence of 0.8 mg/mL of hybrid nanoparticles.

After the synthesis of nanohybrids was completed, we first wanted to apply each technique individually to the HCuS@Cu_2_S@Au–P(NIPAM-*co*-AAm)-PpIX nanohybrids to see what kind of therapeutic properties they would exhibit. Therefore, we first decided to investigate the PDT feature and monitored the decrease in the absorbance of the DPBF trap molecule at approximately 416 nm^28^ by exposing the HCuS@Cu_2_S@Au–P(NIPAM-*co*-AAm)-PpIX nanohybrids to a 630 nm LED for certain periods in the presence of DPBF in PBS/DMSO. As expected, we observed a significant decrease in DPBF absorbance, proving that the PpIX loaded into the nanohybrid structure was effective (Figure 5A). This result also supported the loading of PpIX into HCuS@Cu_2_S@Au–P(NIPAM-*co*-AAm) nanohybrids. Later, the measurement was repeated for HCuS@Cu_2_S@Au–P(NIPAM-*co*-AAm)-PpIX nanohybrids in the presence of the DPBF trap molecule, this time using an 808 nm (1.5 W/cm^2^) laser, and a decrease was observed at an approximately 416 nm wavelength of the trap molecule, due to the formation of singlet oxygen (Figure 5B). However, when compared to the measurement performed with LEDs, this decrease was less as expected due to the absorption characteristics of PpIX.

Second, we examined HCuS@Cu_2_S@Au–P(NIPAM-*co*-AAm)-PpIX nanohybrids’ SDT characteristics. For this purpose, these nanohybrids were also exposed to US for certain periods in the presence of DPBF trap molecules in PBS/DMSO. Again, a decrease in the absorbance of the trap molecule at 416 nm was observed (Figure 5C). This measurement also showed that HCuS@Cu_2_S@Au–P(NIPAM-*co*-AAm)-PpIX nanohybrids produce singlet oxygen with US and have a sonodynamic effect. This further demonstrated again that PpIX was loaded into the nanohybrids.

**Figure 6:**
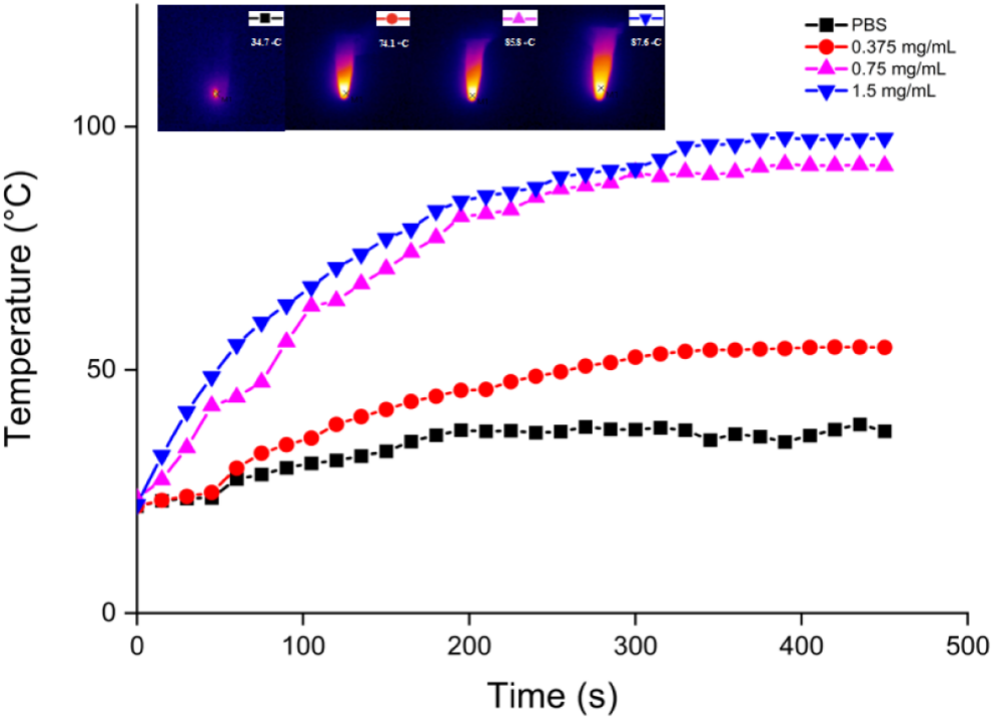
Temperature increases of HCuS@Cu_2_S@Au–P(NIPAM-*co*-AAm)-PpIX nanohybrids at different concentrations in PBS after irradiation with an 808 nm (1.5 W/cm^2^) laser for various times. Inset: thermal camera images of different concentrations of HCuS@Cu_2_S@Au–P(NIPAM-*co*-AAm)-PpIX nanohybrids after irradiation with the 808 nm (1.5 W/cm^2^) laser for 450 s.

After investigating the singlet oxygen generating properties of HCuS@Cu_2_S@Au–P(NIPAM-*co*-AAm)-PpIX nanohybrids, they were treated with an 808 nm (1.5 W/cm^2^) laser to find out whether they had PTT properties. While the temperatures of PBS solutions of nanohybrids exposed to the 808 nm (1.5 W/cm^2^) laser were measured with a thermal camera, the same process was applied to the PBS buffer solution that did not contain nanohybrids (Figure 6). Consequently, compared to the PBS buffer solution, the PBS solutions containing nanohybrids were much more dependent on the amount employed.

**Figure 7:**
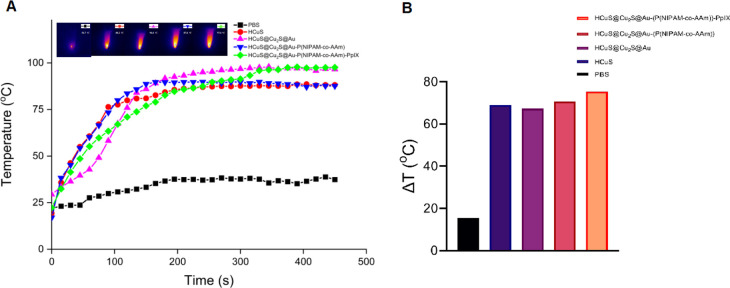
Temperature increases of PBS, HCuS, HCuS@Cu_2_S@Au, HCuS@Cu_2_S@Au–P(NIPAM-*co*-AAm), and HCuS@Cu_2_S@Au–P(NIPAM-*co*-AAm)-PpIX nanohybrids at same concentrations (1.5 mg/mL) after irradiation with an 808 nm (1.5 W/cm^2^) laser for various times. Inset: thermal camera image of nanostructures after laser irradiation for 450 s. (A). Temperature increases of PBS, HCuS, HCuS@Cu_2_S@Au, HCuS@Cu_2_S@Au–P(NIPAM-*co*-AAm), and HCuS@Cu_2_S@Au–P(NIPAM-*co*-AAm)-PpIX nanohybrids (B).

Then, the same experiment was also performed for HCuS, HCuS@Cu_2_S@Au, and polymer-modified HCuS@Cu_2_S@Au–P(NIPAM-*co*-AAm) nanostructures to compare the PTT characteristics of HCuS@Cu_2_S@Au–P(NIPAM-*co*-AAm)-PpIX nanohybrids (Figure 7). As a result, all of these nanostructures exhibit approximately the same PTT characteristics when the temperature versus time graphs at constant concentration are compared. Along with experimental errors, it is typical to see alterations in the heating curves owing to structural differences in HCuS, HCuS@Cu_2_S@Au, HCuS@Cu_2_S@Au–P(NIPAM-*co*-AAm), and HCuS@Cu_2_S@Au–P(NIPAM-*co*-AAm)-PpIX nanostructures.

**Figure 8:**
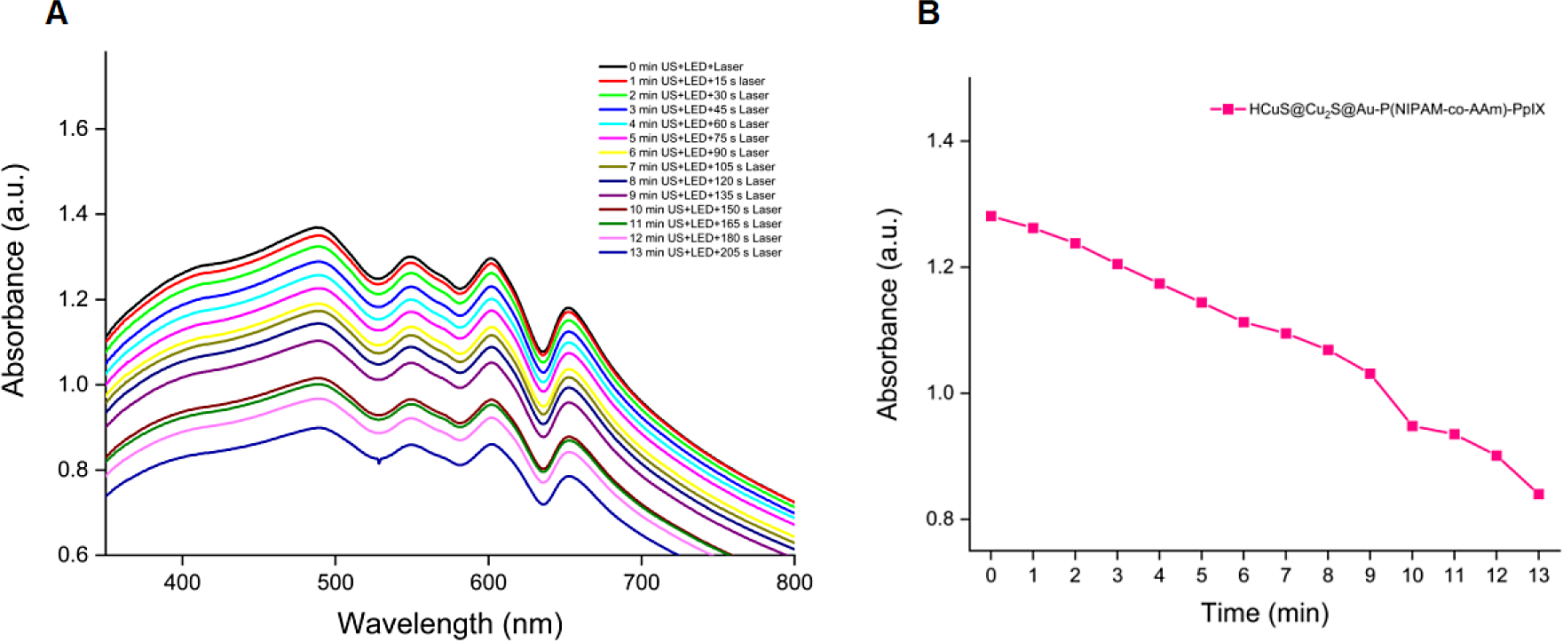
Decrease in the absorbance of DPBF in the presence of 0.8 mg/mL of hybrid nanoparticles at 416 nm in PBS/DMSO after irradiation with 3 MHz US + 630 nm LED + 808 nm (1.5 W/cm^2^) laser (A). The decrease in the maximum absorbance of DPBF at 416 nm over time in the presence of 0.8 mg/mL of hybrid nanoparticles (B).

As the aim of this study, HCuS@Cu_2_S@Au–P(NIPAM-*co*-AAm)-PpIX nanohybrids were exposed to the laser (808 nm, 1.5 W/cm^2^), US (3 MHz), and LED (630 nm) to measure the triple synergistic effects. For this purpose, HCuS@Cu_2_S@Au–P(NIPAM-*co*-AAm)-PpIX nanohybrids (0.8 mg/mL) containing DPBF, which were exposed to both 630 nm LED light and 3 MHz frequency US for 1 min, were treated with the 808 nm laser in the last 15 s of that time (Figures 8A and S5). The absorbance of DPBF decreased steadily over time (Figure 8B). This reduction demonstrates that HCuS@Cu_2_S@Au–P(NIPAM-*co*-AAm)-PpIX nanohybrids produce singlet oxygen with triple application, as well as produced by PDT and SDT alone.

**Figure 9:**
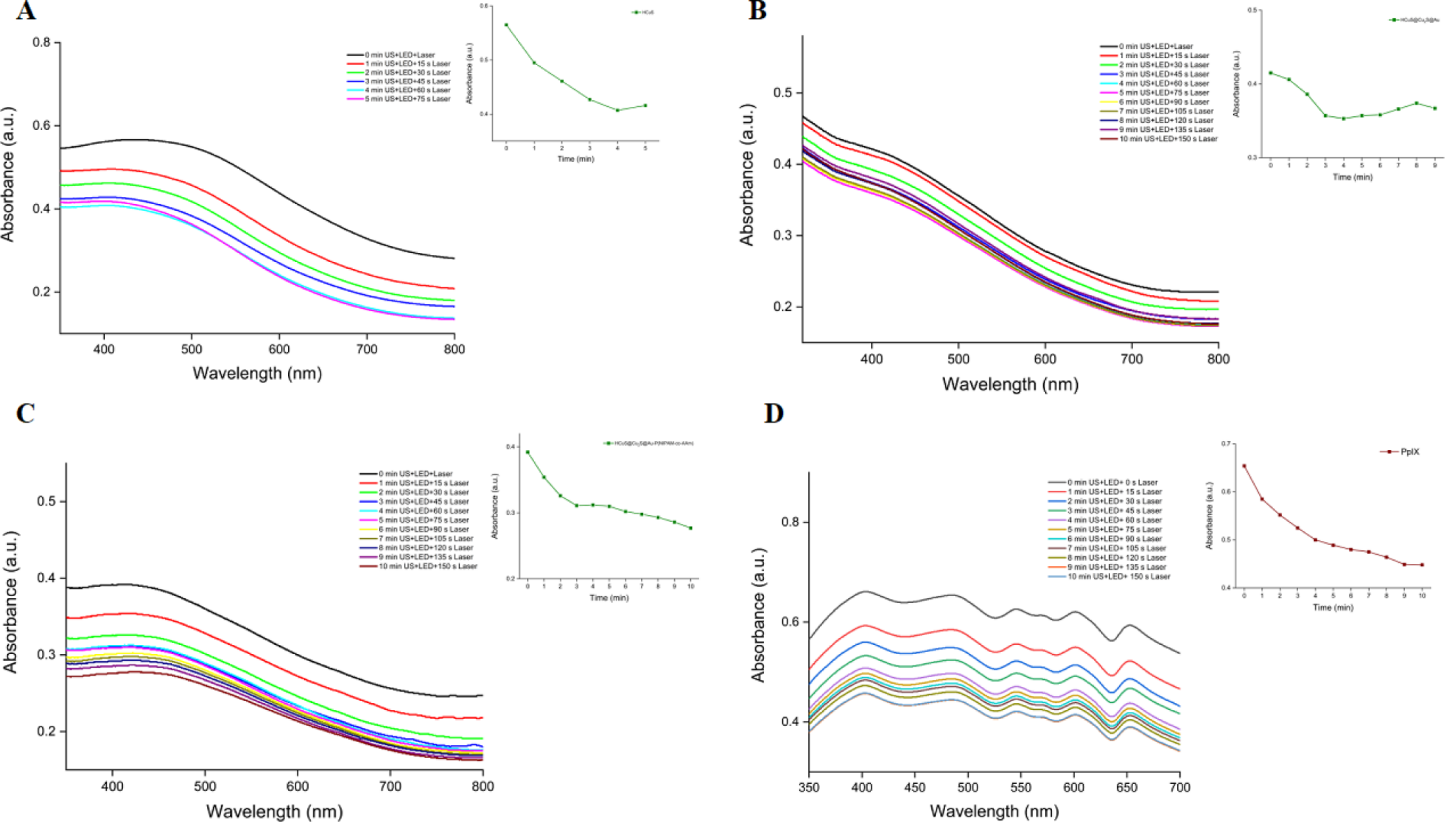
Decrease in the absorbance of DPBF in the presence of 0.8 mg/mL of HCuS (A), HCuS@Cu_2_S@Au (B), HCuS@Cu_2_S@Au–P(NIPAM-*co*-AAm) nanoparticles (C), and PpIX (D) at 416 nm in PBS/DMSO after irradiation with 3 MHz US + 630 nm LED + 808 nm (1.5 W/cm^2^) laser. Insets: graphs show the decrease in the maximum absorbance of DPBF at 416 nm over time in the presence of 0.8 mg/mL of nanoparticles or PpIX.

To compare the triple (US+LED+laser) synergistic effect, samples containing solely HCuS nanoparticles, HCuS@Cu_2_S@Au nanohybrids, polymer-modified HCuS@Cu_2_S@Au–P(NIPAM-*co*-AAm) nanohybrids, and PpIX were also exposed to the LED, laser, and US concurrently in the presence of DPBF as described above. Similarly, singlet oxygen productions were examined by monitoring the absorbance drop of DPBF at 416 nm. As can be seen from the absorbance graphs (Figure 9), each nanoparticle and PpIX separately produced singlet oxygen at certain rates as a result of triple treatment. The other two techniques (US and LED) most likely also contribute somewhat to the generation of singlet oxygen, but the primary reason why HCuS, HCuS@Cu_2_S@Au, and HCuS@Cu_2_S@Au–P(NIPAM-*co*-AAm) nanostructures produce some singlet oxygen is due to the 808 nm (1.5 W/cm^2^) wavelength laser, as it is well-known from the literature that laser irradiation of gold and copper sulfur nanoparticles causes them to produce some singlet oxygen through plasmon resonance.^29^ PpIX itself also produced some singlet oxygen mainly due to US (3 MHz) and LED (630 nm). However, if we compare the singlet oxygen productions of each species using the graphs of the drop in DPBF absorbance at 416 nm, the HCuS@Cu_2_S@Au–P(NIPAM-*co*-AAm)-PpIX nanohybrids’ curve has a much larger decline rate and decrease ratio than those of the others (Figure 8B). In other words, HCuS@Cu_2_S@Au–P(NIPAM-*co*-AAm)-PpIX nanohybrids exhibit stronger PDT activity than any other varieties with a triple application.

**Figure 10:**
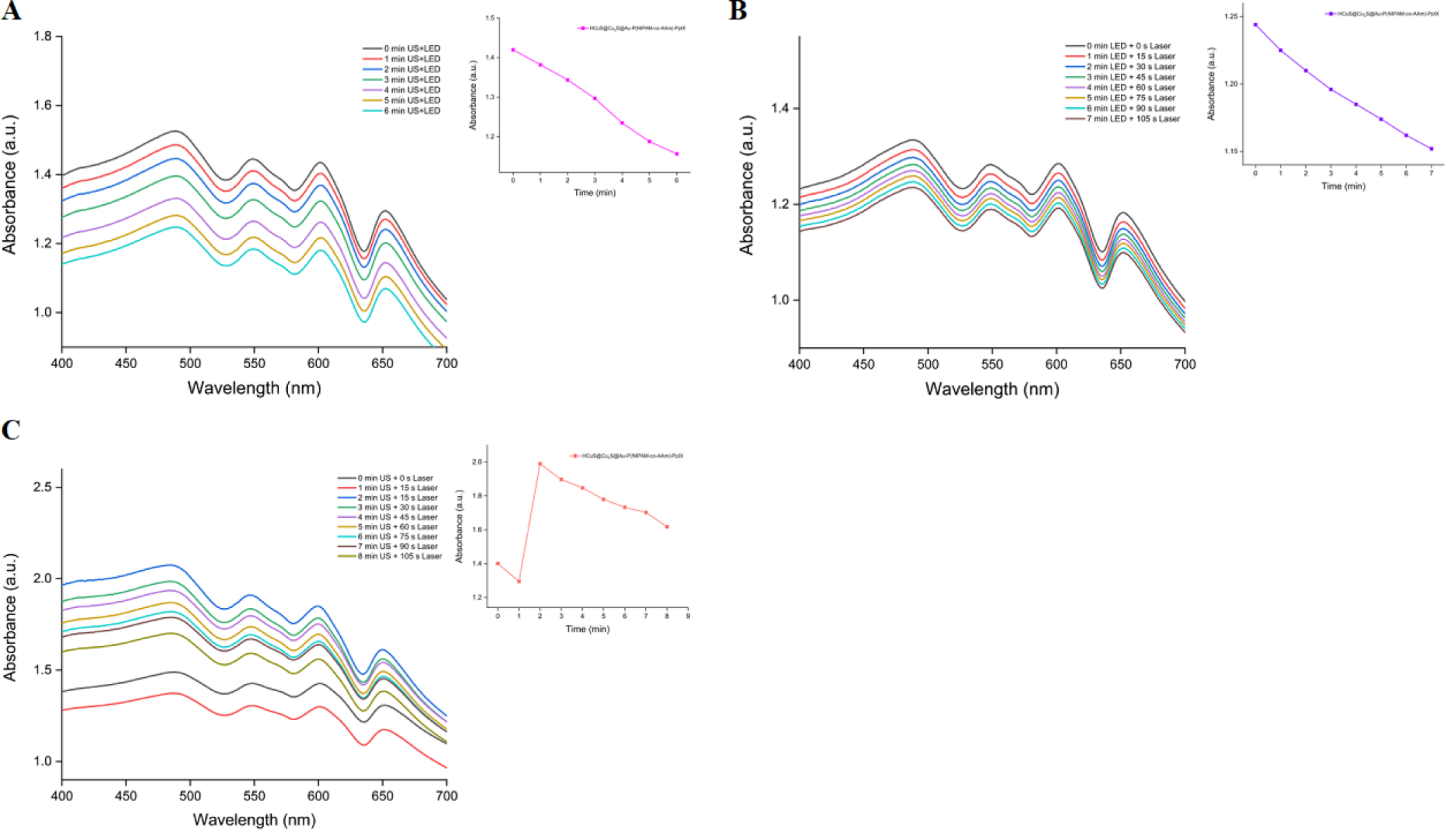
Decrease in the absorbance of DPBF in the presence of 0.8 mg/mL of hybrid nanoparticles at 416 nm in PBS/DMSO after irradiation with 3 MHz US + 630 nm LED (A), 630 nm LED + 808 nm (1.5 W/cm^2^) laser (B), and 3 MHz US + 808 nm (1.5 W/cm^2^) laser (C). Insets: the decrease in the absorbance of DPBF in the presence of 0.8 mg/mL of hybrid nanoparticles at 416 nm over times.

Additionally, the HCuS@Cu_2_S@Au–P(NIPAM-*co*-AAm)-PpIX nanohybrids were exposed to US, LED, and laser in dual combinations to gain a better understanding of the effects of the methods used on the PDT properties of the nanohybrids. So, HCuS@Cu_2_S@Au–P(NIPAM-*co*-AAm)-PpIX nanohybrids were exposed to US, LED, and laser in the presence of DPBF in dual combinations as (US+LED), (LED+laser), and (US+laser) and the decrease in the absorbance of the DPBF trap molecule at 416 nm was monitored (Figure 10). Thus, dual combinations of applications were also compared. Therefore, it can be concluded that the PDT effects are quite comparable to one another based on the highly close negative values of the slopes of the declining curves in the (LED+laser) and (US+LED) applications. On the other hand, while a comparable decline is noted in the (US+laser) application, the decrease is somewhat less and PDT’s efficacy is lower. This is primarily because the 630 nm LED provides the largest contribution to PpIX’s production of singlet oxygen. In addition, an intriguing circumstance noted in the context of (US+laser) application is that absorbance abruptly rises following the first minute of application and then steadily declines following the second minute. This can be explained by the rapid release of PpIX and the sudden increase in absorptivity as a result of both the sonic effect and the effect of the sudden heating caused by the laser on the copolymer at the first moment of dual application. However, this effect was not observed in other dual and triple applications. One possible explanation for this could be that the released PpIX from nanohybrids may not have the same releasing effect in other dual and triple applications. Consequently, while the singlet oxygen production and the PDT effect of nanohybrids were determined in all dual applications, the PDT effect observed in the triple application was still greater than all dual applications.

**Figure 11:**
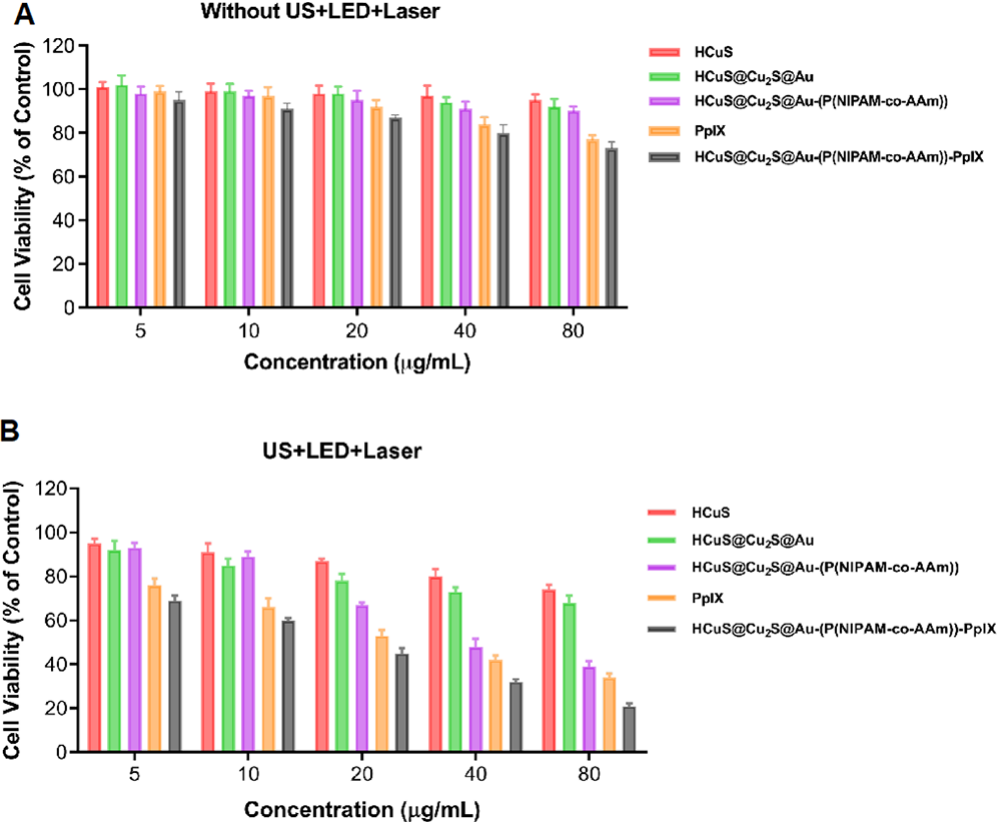
Concentration-dependent proliferation inhibition of MDA-MB-231 cells treated with HCuS, HCuS@Cu_2_S@Au, HCuS@Cu_2_S@Au-(P(NIPAM-*co*-AAm)), PpIX, and HCuS@Cu_2_S@Au-(P(NIPAM-*co*-AAm))-PpIX nanohybrids only. Cell viability was evaluated using the XTT test, and the results are presented as mean ± SD in triplicate (A). Concentration-dependent proliferation inhibition of MDA-MB-231 cells treated with HCuS, HCuS@Cu_2_S@Au, HCuS@Cu_2_S@Au-(P(NIPAM-*co*-AAm)), PpIX, and HCuS@Cu_2_S@Au-(P(NIPAM-*co*-AAm))-PpIX nanohybrids after exposure to LED+US+laser. Cell viability was evaluated using the XTT test, and the results are presented as mean ± SD in triplicate (B).

**Figure 12:**
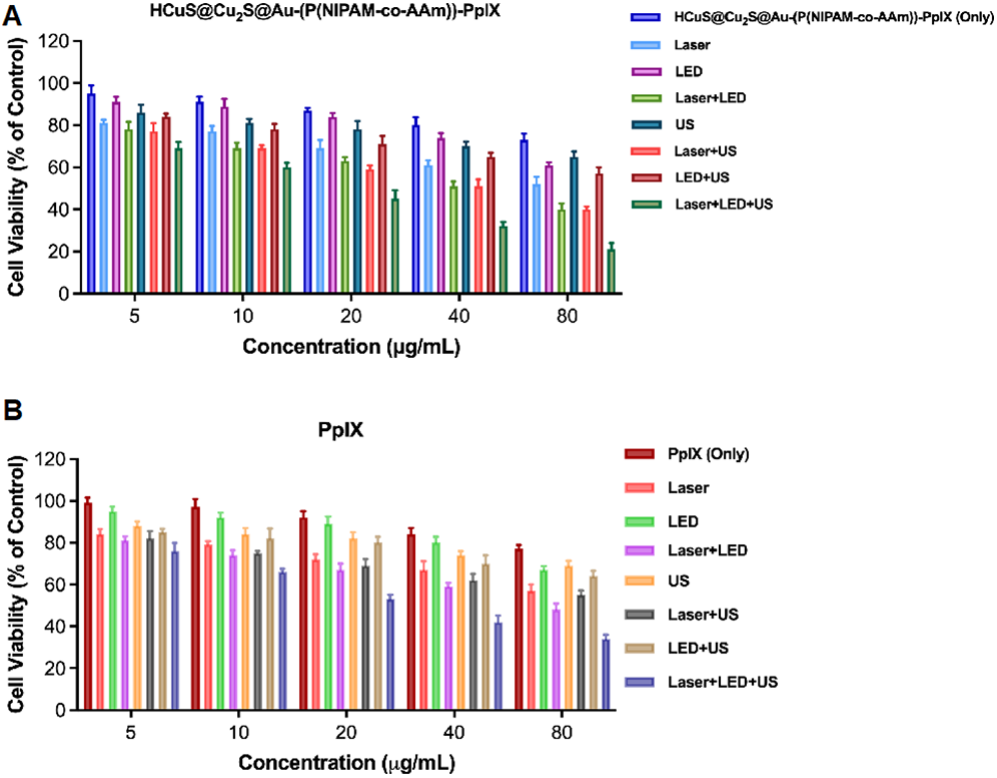
Concentration-dependent proliferation inhibition of MDA-MB-231 cells treated with PpIX and HCuS@Cu_2_S@Au-(P(NIPAM-*co*-AAm))-PpIX nanohybrids only and after exposure to laser, LED, US, or their combinations. Cell viability was evaluated using the XTT test and the results are presented as mean ± SD in triplicate.

After verifying the HCuS@Cu_2_S@Au-(P(NIPAM-*co*-AAm))-PpIX nanohybrids’ PDT, SDT, and PTT characteristics, all components involved in the study, namely HCuS, HCuS@Cu_2_S@Au, HCuS@Cu_2_S@Au-(P(NIPAM-*co*-AAm)), PpIX, and HCuS@Cu_2_S@Au-(P(NIPAM-*co*-AAm))-PpIX (only/with or without LED, laser, and US or their combinations), have been tested for their cytotoxicity in MDA-MB-231 cells. The cells were exposed to different concentrations of HCuS, HCuS@Cu_2_S@Au, HCuS@Cu_2_S@Au-(P(NIPAM-*co*-AAm)), PpIX, and HCuS@Cu_2_S@Au-(P(NIPAM-*co*-AAm))-PpIX and their antiproliferative effects were evaluated with the XTT test. As presented in Figure 11A, no cytotoxicity was observed in the HCuS, HCuS@Cu_2_S@Au, HCuS@Cu_2_S@Au-(P(NIPAM-*co*-AAm)), PpIX, and HCuS@Cu_2_S@Au-(P(NIPAM-*co*-AAm))-PpIX only groups. However, as shown in Figure 12B, after the treatment of PpIX, it has been observed that laser+LED, laser+US, and LED+US applications (laser+LED > laser+US > LED+US) have more toxic effects compared to laser, LED, and US applications alone. As expected, after PpIX application, the highest toxicity was obtained in the laser+LED+US group in a concentration-dependent manner.

Figure 12A also shows the toxic effects of HCuS@Cu_2_S@Au-(P(NIPAM-*co*-AAm))-PpIX nanoparticles alone, LED, laser, US, or their combination on MDA-MB-231 cells. Similar to PpIX, treatment with HCuS@Cu_2_S@Au-(P(NIPAM-*co*-AAm))-PpIX alone did not show a significant toxic effect on cancer cells. However, it has been determined that the application of laser+LED+US increases the toxicity of cancer cells compared to alone laser, LED, US, or their combinations. Moreover, it was evaluated that the application of HCuS@Cu_2_S@Au-(P(NIPAM-*co*-AAm))-PpIX alone or in the form of laser, LED, US, or their combination has more toxic effects than PpIX. As seen in Figure 11B, the LED+US+laser combination showed a concentration-dependent toxic effect in all groups, but the highest toxicity was found to be in the HCuS@Cu_2_S@Au-(P(NIPAM-*co*-AAm))-PpIX group.

**Figure 13:**
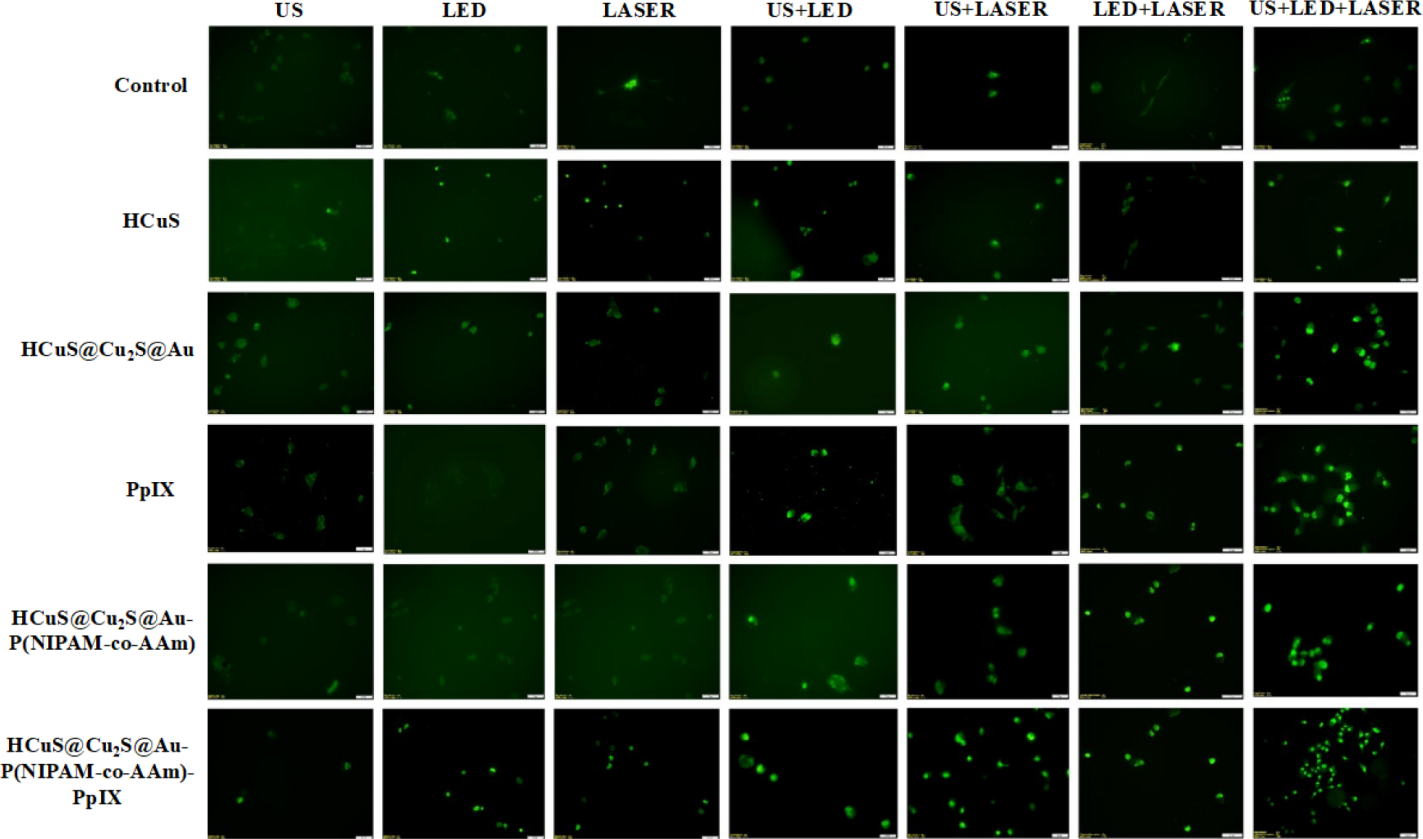
Fluorescence microscopic images of TUNEL staining of MDA-MB-231 cells treated with different concentrations of HCuS, HCuS@Cu_2_S@Au, PpIX, HCuS@Cu_2_S@Au–P(NIPAM-*co*-AAm), and HCuS@Cu_2_S@Au–P(NIPAM-*co*-AAm)-PpIX, and exposed to US (3 MHz), LED (690 nm), laser (808 nm, 1 W/cm^2^), US+LED, US+laser, LED+laser, and US+LED+laser.

In the final step of the study, the In Situ Cell Death Detection Kit, Fluorescein was used to evaluate cell death by apoptosis (TUNEL kit, Roche, Germany). All procedures were performed as specified by the manufacturer. So, MDA-MB-231 cells undergoing apoptosis were marked with green fluorescence by TUNEL staining, resulting in a fluorescence microscopic image generated by the green glow of apoptotic cell nuclei (Figure 13). All control groups had the least number of apoptotic cells, as expected. In general, apoptosis increased from HCuS nanoparticles to HCuS@Cu_2_S@Au–P(NIPAM-*co*-AAm)-PpIX nanohybrids in all single, dual, and triple applications. Additionally, the highest apoptotic cell population in all applications was observed for CuS@Cu_2_S@Au–P(NIPAM-*co*-AAm)-PpIX nanohybrids. Moreover, among all species and applications examined, the laser+US+LED triple application of HCuS@Cu_2_S@Au–P(NIPAM-*co*-AAm)-PpIX nanohybrids resulted in the highest number of apoptotic cells (Figure 13, last column). This result once again supported that HCuS@Cu_2_S@Au–P(NIPAM-*co*-AAm)-PpIX nanohybrids responded and worked in the triple application.

## Conclusions

Combining the advantages of triple therapeutic techniques into an all-in-one approach is a smart idea. As a result, thermopolymer-modified and PpIX-loaded hollow HCuS@Cu_2_S@Au–P(NIPAM-*co*-AAm)-PpIX nanohybrids successfully fulfilled their intended functions. The all-in-one design of HCuS@Cu_2_S@Au–P(NIPAM-*co*-AAm)-PpIX nanohybrids is further supported by the fact that the triple application (laser, US, and LED) in in vitro cell investigations is more effective than all other single and dual applications. Of course, the all-in-one idea could be enhanced with the addition of a wider variety of therapeutic techniques and targeting systems. Thus, our laboratory is actively researching new designs that will develop the all-in-one idea. As a result, we may not have been able to come up with a definitive treatment for cancer, but we have developed a very successful novel noninvasive nanohybrid system that could inspire many future studies in this field. At some point, this noninvasive strategy and nanohybrids could serve as a blueprint for future cutting-edge concepts, paving the way for creating novel and highly efficacious designs.

## Experimental Section

### Synthesis of Hollow CuS (HCuS) Nanospheres

HCuS nanospheres were synthesized according to the previously reported method in the literature reference. 100 μL of CuCl_2_ (0.5 M) was added to 25 mL of deionized water containing 0.24 g of PVP-K30 under magnetic stirring at room temperature. Next, a solution of NaOH (25 mL, pH = 9.0) was introduced, followed by the addition of N_2_H_4_ ·H_2_O (6.4 μL, 50%) to produce a bright-yellow suspension of Cu_2_O spheres. After a duration of 5 min, an aqueous solution of Na_2_S (200 μL, 320 mg mL^–1^) was incorporated into the suspension. The mixture was then heated for 2 h at 60 °C. Subsequently, the HCuS nanospheres were subjected to centrifugation at 12 000 rpm for a span of 8 min and were rinsed thrice with deionized water and ethanol. Finally, the HCuS nanospheres were dispersed in ethanol (20 mL).

### Synthesis of Hollow CuS@Cu_2_S@Au (HCuS@Cu_2_S@Au) Nanohybrids

The HCuS@Cu_2_S@Au nanohybrids were one-step synthesized according to the previously reported literature with some modifications. 1.0 mL of HCuS suspension was dispersed in 1.2 mL of ethanol. This was followed by addition of 0.01 g of PVP-K30. After stirring for 30 min, an aqueous solution of HAuCl_4_·3H_2_O (0.3 mM, 6.4 mL) was added, and the mixture was stirred for 10 min. After that, 0.5 mL of NaBH_4_ (3 mM) was added, and the mixture was stirred for another 30 min. The products were collected by centrifugation at 12 000 rpm for 10 min and washed several times with ethanol.

### Synthesis of Poly(*N*-isopropylacrylamide-*co*-acrylamide) (P(NIPAM-*co*-AAm) Thermosensitive Copolymer

P(NIPAM-*co*-AAm) was prepared by RAFT (reversible addition–fragmentation chain transfer) polymerization according to the literature. 3g of *N*-isopropylacrylamide (NIPAAm), 0.3063 g of acrylamide (AAm), 60 μL of chain transfer agent 2-mercaptoethanol (2-ME), and 9 mg of initiator 4,4′-azobis(4-cyanopentanoic acid) (ACPA) were dissolved in 10 mL of methanol. The solution was degassed by bubbling with argon for 45 min. After the mixture was continuously stirred at 70 °C for 24 h, the resulting product was precipitated out by the addition of diethyl ether. The product was purified by repeated precipitation in diethyl ether and then dried in vacuum.

### Synthesis of P(NIPAM-*co*-AAm) Copolymer-Modified HCuS@Cu_2_S@Au–P(NIPAM-*co*-AAm) Nanohybrids

The HCuS@Cu_2_S@Au nanohybrids were dispersed in H_2_O (0.3 mg/mL). Then, 5 mL of P(NIPAAm-*co*-AAm) solution (0.04 mg/mL) was added into the aqueous dispersion of HCuS@Cu_2_S@Au. The mixture was stirred for 24 h at room temperature. After that, the product was washed several times with water to remove the unreacted polymer.

### Synthesis of PpIX-Loaded HCuS@Cu_2_S@Au–P(NIPAM-*co*-AAm)-PpIX Nanohybrids

0.645 mg portion of HCuS@Cu_2_S@Au–P(NIPAM-*co*-AAm) was dispersed in 1 mL of distilled water, and then the dispersion was added into the PpIX solution (DMSO/PBS). The mixtures were stirred at 40 °C for 24 h under dark conditions. The precipitate was separated by centrifugation and washed with distilled water several times until the supernatant became colorless.

### Study of SDT Properties of HCuS@Cu_2_S@Au–P(NIPAM-*co*-AAm)-PpIX Nanohybrids

The singlet oxygen generation ability of HCuS@Cu_2_S@Au–P(NIPAM-*co*-AAm)-PpIX was assessed using 1,3-diphenylisobenzofuran (DPBF) as an indicator. Briefly, 100 μL of HCuS@Cu_2_S@Au–P(NIPAM-*co*-AAm)-PpIX dispersion (0.8 mg/mL) in PBS (pH 7.3) was mixed with 25 μL of DPBF, and then the dispersion was transferred into a cuvette. The mixture was irradiated by US (3 MHz, 60% duty cycle, 1.0 W/cm^2^) with an interval of 1 min in dark conditions. The intensity of DPBF was recorded by a UV–vis spectrophotometer. The reaction of DPBF with ROS results in the decay of its absorption intensity of DPBF in the UV–VIS curve at about 416 nm.

### Study of PDT Properties of HCuS@Cu_2_S@Au–P(NIPAM-*co*-AAm)-PpIX Nanohybrids

Singlet oxygen generation potential of HCuS@Cu_2_S@Au–P(NIPAM-*co*-AAm)-PpIX nanohybrids was investigated initially using a DPBF trap molecule. Briefly, 100 μL of HCuS@Cu_2_S@Au–P(NIPAM-*co*-AAm)-PpIX dispersion (0.8 mg/mL) in PBS (pH 7.3) was mixed with 25 μL of DPBF, and then transferred into a cuvette. The mixture was kept in a dark environment. The mixture was irradiated with a 630 nm LED for a certain period and the UV–vis absorption spectra were recorded. The ^1^O_2_ generation was evaluated by the decay of the UV–vis absorption intensity decrease of DPBF at about 416 nm.

### Study of PTT Properties of HCuS@Cu_2_S@Au–P(NIPAM-*co*-AAm)-PpIX Nanohybrids

A thermal camera was used to characterize the photothermal performance of HCuS@Cu_2_S@Au–P(NIPAM-*co*-AAm)-PpIX nanohybrids by recording the temperature changes during laser irradiation every 15 s. 200 μL of HCuS@Cu_2_S@Au–P(NIPAM-*co*-AAm)-PpIX nanohybrid dispersions in PBS (pH 7.3) with different amounts of HCuS@Cu_2_S@Au–P(NIPAM-*co*-AAm)-PpIX nanohybrids (0.375 mg/mL, 0.75 mg/mL, and 1.5 mg/mL) was illuminated by an 808 nm laser at a power density of 1.5 W/cm^2^ for 450 s. The temperature changes in PBS (pH 7.3) dispersions were monitored with a thermal camera (Testo IR). As a control experiment, only PBS (pH 7.3) solution was exposed to laser light for 450 s, and temperature changes were also monitored by a thermal camera every 15 s.

### Determining the SDT–PDT–PTT Triple Synergistic Effect of HCuS@Cu_2_S@Au–P(NIPAM-*co*-AAm)-PpIX Nanohybrids

SDT–PDT–PTT combination potential of HCuS@Cu_2_S@Au–P(NIPAM-*co*-AAm)-PpIX nanohybrids was investigated using DPBF. 100 μL aliquot of HCuS@Cu_2_S@Au–P(NIPAM-*co*-AAm)-PpIX dispersion (0.8 mg/mL) in PBS (pH 7.3) was mixed with 25 μL of DPBF. The mixture was irradiated with a 630 nm LED and 3 MHz 1 W/cm^2^ US for 1 min and 808 nm 1.5 W/cm^2^ laser for 15 s, simultaneously. The ^1^O_2_ generation was assessed by the decay of the UV–vis absorption intensity decrease of DPBF at about 416 nm.

### Cell Lines and Cell Culture

Human breast cancer cells MDA-MB-231 (HTB-26) were provided from the American Type Culture Collection (ATCC, USA). A mixture of DMEM (Sigma-Aldrich), fetal bovine serum (FBS) (Gibco, Thermo Fisher Scientific), and penicillin/streptomycin (Gibco, ThermoFisher Scientific) was prepared to culture cells. The cells were grown under sterile conditions at 37 °C within a 5% CO_2_ humidified incubator. HCuS, HCuS@Cu_2_S@Au, HCuS@Cu_2_S@Au-(P(NIPAM-*co*-AAm)), PpIX, and HCuS@Cu_2_S@Au-(P(NIPAM-*co*-AAm))-PpIX nanohybrids were dissolved in DMSO and this stock was further diluted with the DMEM mixture before treatment with a final percentage of DMSO not exceeding 0.1%. The control cells were also treated with DMEM with 0.1% DMSO.

### Cytotoxicity Assay

The effect of various concentrations of HCuS, HCuS@Cu_2_S@Au, HCuS@Cu_2_S@Au-(P(NIPAM-*co*-AAm)), PpIX, and HCuS@Cu_2_S@Au-(P(NIPAM-*co*-AAm))-PpIX nanohybrids on MDA-MB-231 cell proliferation (only or after treatment with laser, LED, US, or their combinations) was assessed with the XTT (2,3-bis(2-methoxy-4-nitro-5-sulfophenyl)-2*H*-tetrazolium-5-carboxanilide) (Roche) assay. The cells were seeded into sterile 96 plates with 1 × 10^4^ cells in each well before applications and incubated overnight for the cells to adhere to the plate bottoms. At the end of the incubation period, the cells were exposed to HCuS, HCuS@Cu_2_S@Au, HCuS@Cu_2_S@Au-(P(NIPAM-*co*-AAm)), PpIX, and HCuS@Cu_2_S@Au-(P(NIPAM-*co*-AAm))-PpIX nanohybrids at various concentrations (5,10, 20, 40, and 80 μg/mL) for 24 h. The next day, the DMEM mixtures were removed, the wells were washed three times with PBS, and 100 mL of fresh DMEM was added to the wells. After laser, LED, US, or their combinations (LED (1 min)+laser (15 s); LED (1 min)+US (1 min); US (1 min)+laser (15 s); LED (1 min)+US (1 min)+laser (15 s)) application, the cells were reincubated for 24 h. Then, the media containing various concentrations of HCuS, HCuS@Cu_2_S@Au, HCuS@Cu_2_S@Au-(P(NIPAM-*co*-AAm)), PpIX, and HCuS@Cu_2_S@Au-(P(NIPAM-*co*-AAm))-PpIX were then aspirated, and the wells were washed with PBS. The DMEM and XTT mixture was added to each well and incubated for another 4 h. Lastly, the absorbance was determined using an ELISA microplate reader (Thermo) at 450 nm, and the cell viability was determined as a viable cell amount percent compared to control cells.

### Fluorescence Microscopy Analysis

In Situ Cell Death Detection Kit, Fluorescein was used to evaluate cell death by apoptosis (TUNEL kit, Roche, Germany). The MDA-MB-231 breast cancer cell line was seeded on sterile coverslips placed in 6-well plates, with 100 000 cells in each. Laser, LED, US, and their combinations (LED (1 min)+laser (15 s); LED (1 min)+US (1 min); US (1 min)+laser (15 s); LED (1 min)+US (1 min)+laser (15 s)) were applied to the MDA-MB-231 breast cancer cells grown on sterile coverslips exposed to HCuS, HCuS@Cu_2_S@Au, HCuS@Cu_2_S@Au-(P(NIPAM-*co*-AAm)), PpIX, and HCuS@Cu_2_S@Au-(P(NIPAM-*co*-AAm))-PpIX nanohybrids at the same concentrations as mentioned in the Cytotoxicity Assay section. The cells from which the medium was removed were washed twice with PBS. Then, they were fixed in freshly prepared 4% paraformaldehyde/PBS (Sigma, Germany), pH 7.4, at room temperature for 60 min and washed with PBS. The cells were permeabilized with freshly prepared Triton X-100 in 1% sodium citrate for 2 min at 2–8 °C. 100 μL of the label solution in the kit was taken, and 50 μL was applied to the negative controls. The enzyme solution from the kit and the label solution were mixed and incubated. Before applying the TUNEL mixture prepared to detect DNA breaks in positive controls, they were kept in micrococcal nuclease/DNase 1 recombinant solution at 15–25 °C for 10 min (3000U/ml–3U/ml in 50 mM Tris-HCl, pH 7.5, 1 mg/mL BSA). Then, 50 μL of TUNEL mix solution (label and enzyme solution mixture) was applied to the cells, passed through PBS twice on each sample, and incubated for 60 min in a moist, dark environment at 37 °C. They were rewashed in PBS-Triton-X for three changes. Following three washes in PBS-Triton-X 100, sections were evaluated using a fluorescence microscope (Olympus BX51). All steps of the immunostaining process were applied to the negative control sections without the primary antibody incubation. Pictures from the convenient fields of view were taken by Olympus BX51 (Tokyo, Japan).

## Supporting Information Available

Size distribution of HCuS and HCuS@Cu2S@Au nanoparticles (Figure S1); N_2_ adsorption–desorption isotherms and pore size distributons of HCuS and HCuS@Cu2S@Au nanoparticles (Figure S2); ^1^H-NMR spectrum of P(NIPAM-*co*-AAm) (Figure S3); size distribution of HCuS@Cu_2_S@Au–P(NIPAM-*co*-AAm) and HCuS@Cu_2_S@Au–P(NIPAM-*co*-AAm)-PpIX nanohybrids (Figure S4); average particle size and zeta potentials of nanoparticles (Table S1); experimental setup photo (Figure S5) (PDF)
