# Supplementary material for: All-in-One Nanohybrids Combining Sonodynamic Photodynamic and Photothermal Therapies
Source: ACS Appl Mater Interfaces. 2024 Aug 13;16(33):43387–99. doi: 10.1021/acsami.4c09715 (PMC11345719; doi:10.1021/acsami.4c09715)
Supplement: Supplementary file 1 — am4c09715_si_001.pdf [file am4c09715_si_001.pdf]

Supporting Information

All-in-one Nanohybrids Combining Sonodynamic Photodynamic and Photothermal Therapies

Dilşad Taydaş,[1] Muhammed Emre Özler,[1] Mustafa Ergül,[2] Zeynep Deniz Şahin İnan,[3] Fazlı Sözmen[1,] *

[1] Nanotechnology Engineering Department, Faculty of Engineering, Sivas Cumhuriyet University, 58140 Sivas, Türkiye

[2] Biochemistry Department, Faculty of Pharmacy, Sivas Cumhuriyet University, 58140 Sivas, Türkiye

[3] Histology and Embryology Department, Faculty of Medicine, Sivas Cumhuriyet University, 58140 Sivas, Türkiye

*Corresponding Author E-mail: fsozmen@cumhuriyet.edu.tr

A

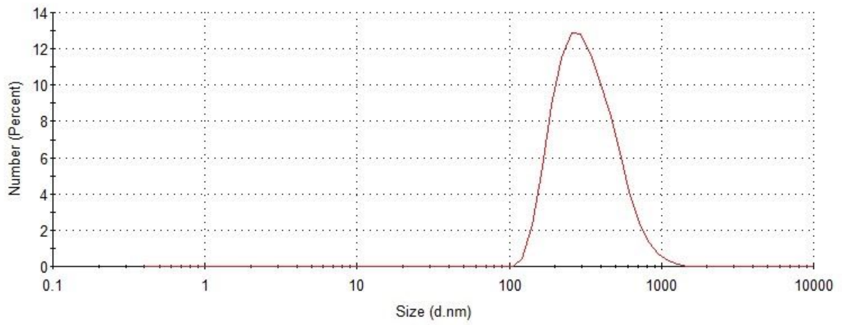

B

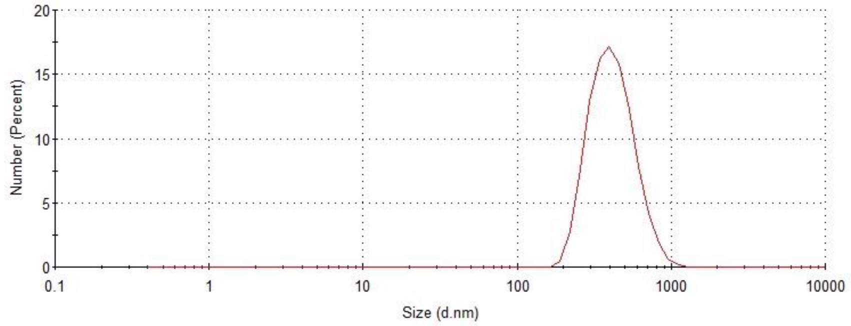

Figure S1. Size distribution of HCuS nanoparticles (A), HCuS@Cu_2S@Au nanohybrids (B).

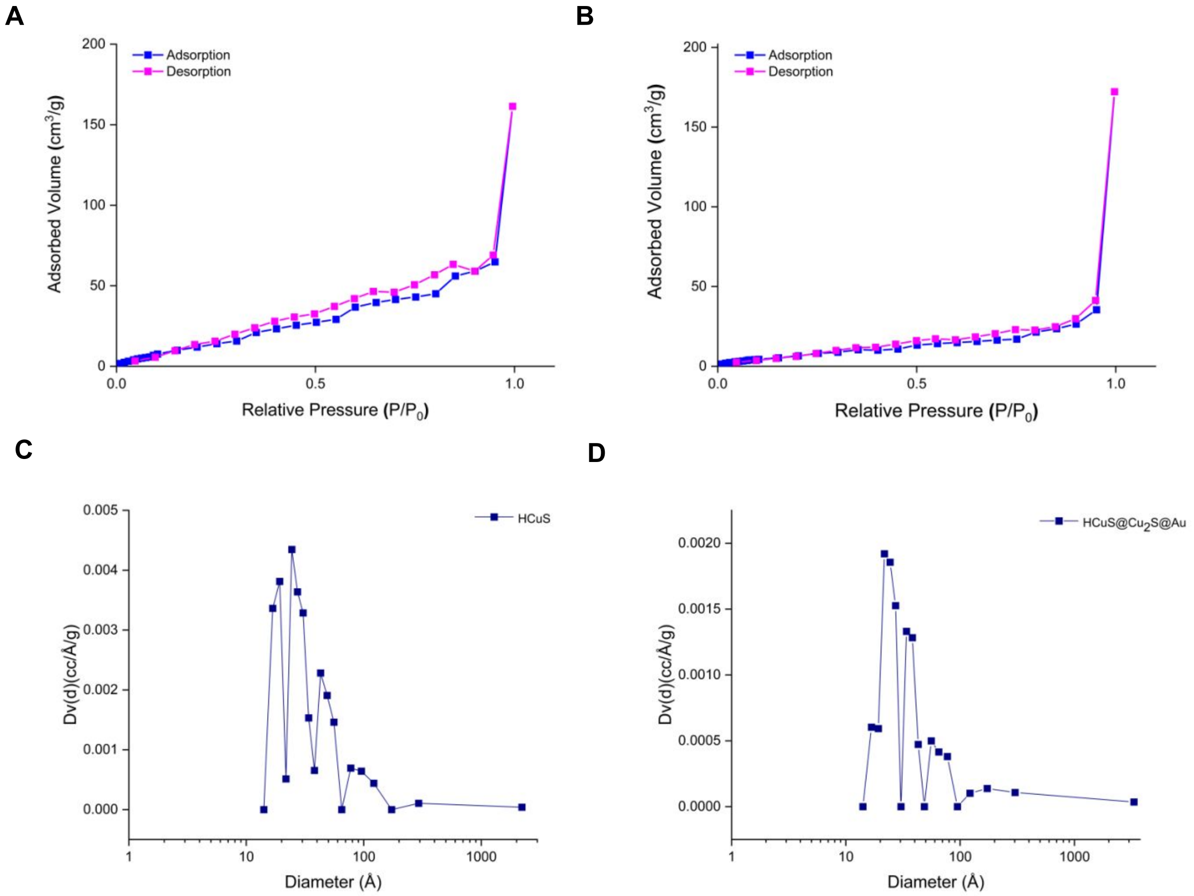

Figure S2. N_2 adsorption-desorption isotherms of HCuS nanoparticles (A), HCuS@Cu_2S@Au nanohybrids (B). Pore size distribution of HCuS nanoparticles (C), HCuS@Cu_2S@Au nanohybrids (D).

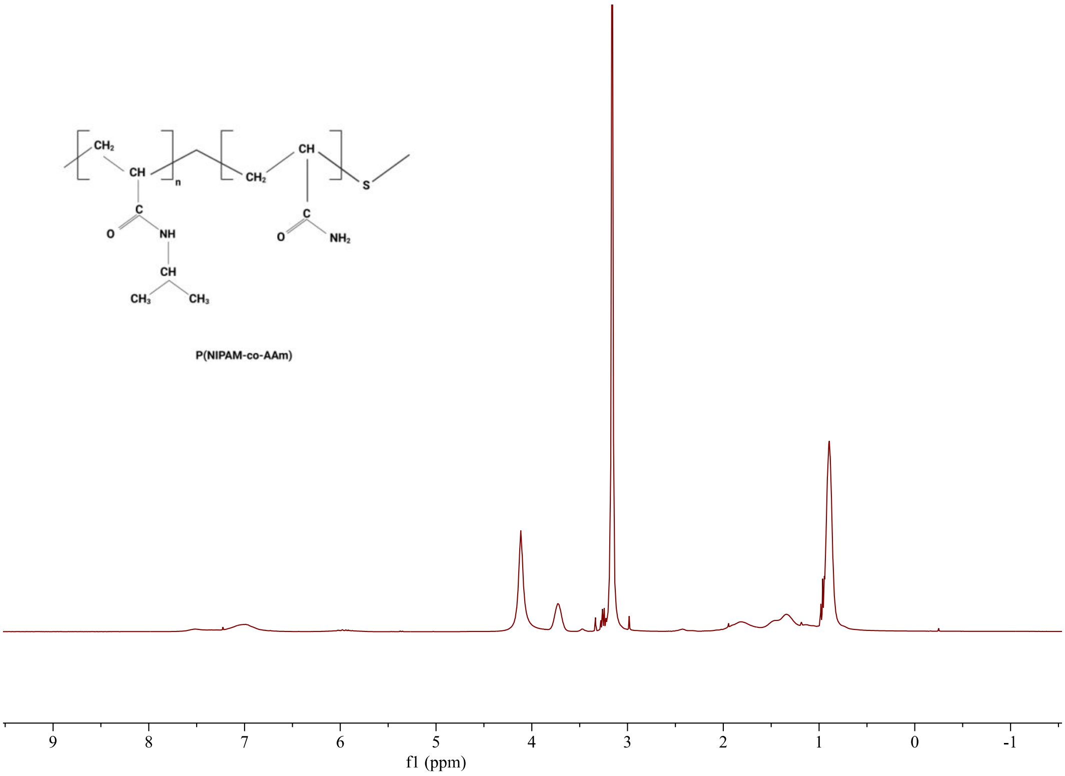

Figure S3. ^{1}H-NMR spectrum of P(NIPAM-co-AAm) copolymer in $CDCl_3$.

A

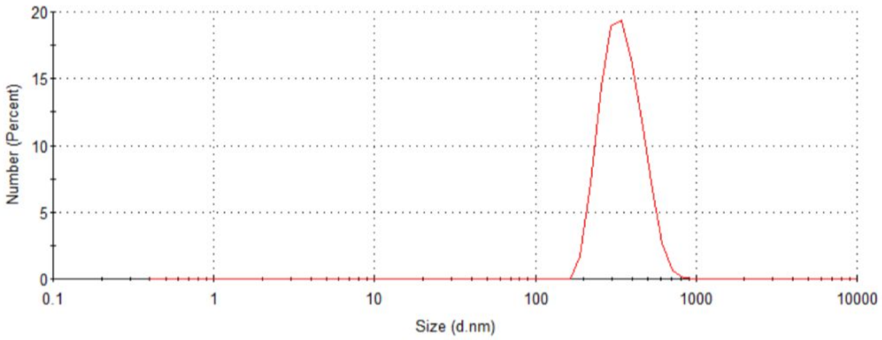

B

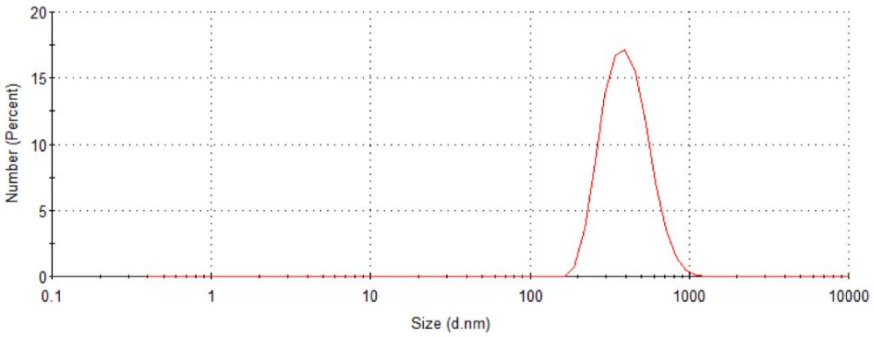

Figure S4. Size distribution of HCuS@Cu_2S@Au-P(NIPAM-co-AAm) nanohybrids (A), HCuS@Cu_2S@Au-P(NIPAM-co-AAm)-PpIX nanohybrids (B).

Nanoparticle	Average Particle Size (nm)	Zeta Potential (mV)
HCuS	343	-12.25
HCuS@Cu_2S@Au	427	-17.06
HCuS@Cu_2S@Au-P(NIPAM-co-AAm)	354	-10.26
HCuS@Cu_2S@Au-P(NIPAM-co-AAm)-PpIX	414	-9.996

Table S1. Size distribution and zeta potentials of nanostructures.

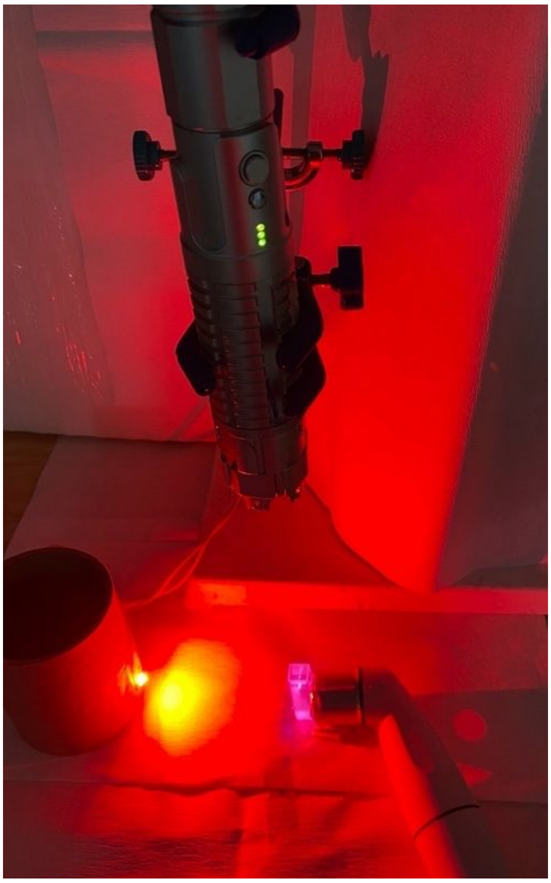

Figure S5. The digital photo of the experimental setup for the SDT-PDT-PTT triple application.
